# Supplementary figures and images for: Synthetic STARR-seq reveals how DNA shape and sequence modulate transcriptional output and noise
Source: PLoS Genet. 2018 Nov 14;14(11):e1007793. doi: 10.1371/journal.pgen.1007793 (PMC6261644; doi:10.1371/journal.pgen.1007793)

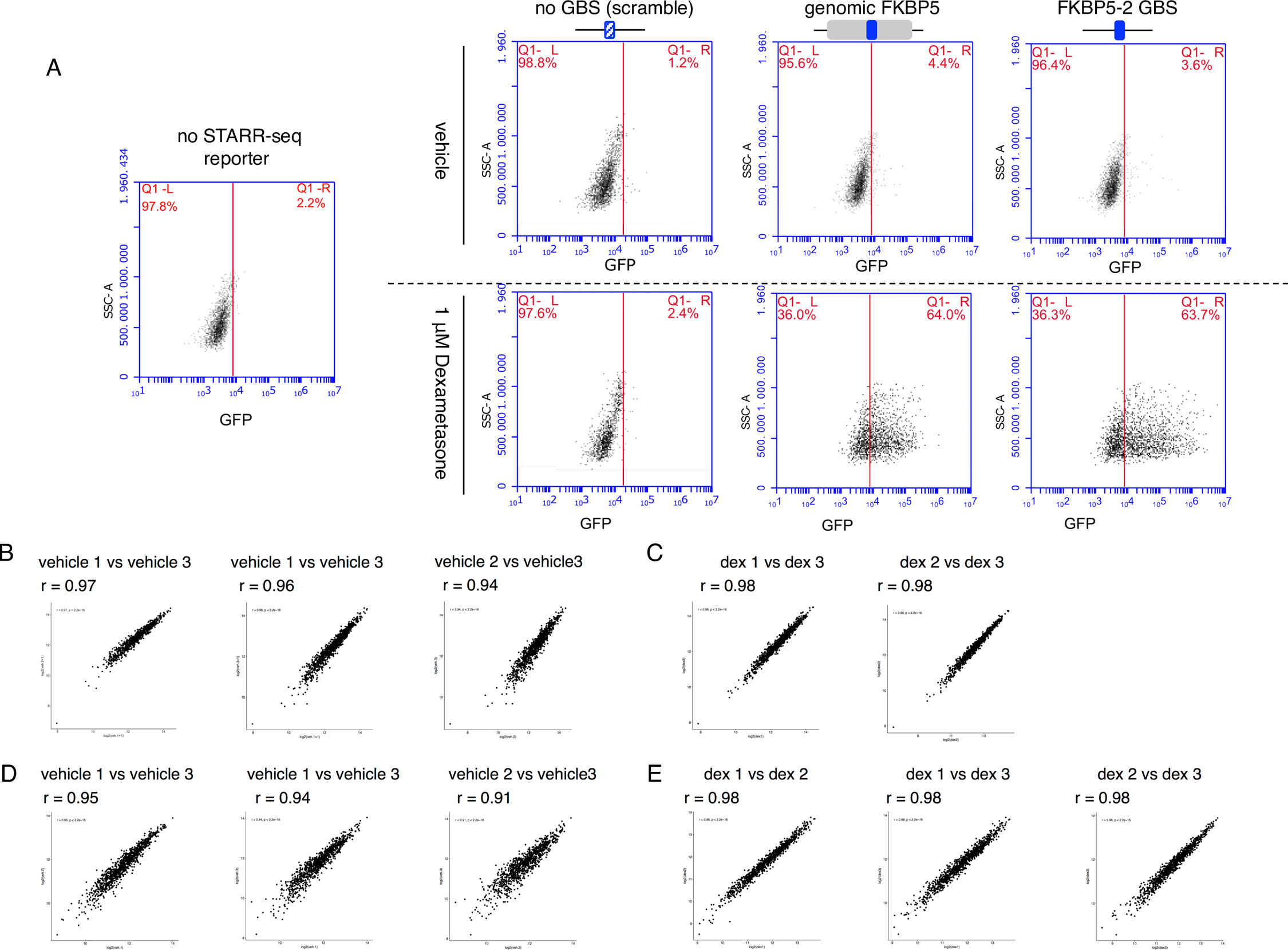

Supplement: S1 Fig — (a) Analysis of individual enhancer variants as indicated by flow cytometry showing the side scatter (SSC-A) versus GFP signal for individual mCherry-positive cells. Left: no STARR-seq construct. Right-Top: ethanol, vehicle, treated cells; Right-Bottom: Cells treated overnight with 1μM dexamethasone. Numbers in red indicate the percentage of GFP+ (top right side) and GFP- (top left side) cells respectively. Red vertical line demarcates the threshold for being called GFP+. (b) RNA-seq correlation plots for biological replicates of vehicle-treated cells transfected with the GBS-flank library (Cgt flank library). (c) Same as (b) except for biological replicates of dexamethasone-treated cells (4h 1μM). (d) RNA-seq correlation plots for biological replicates of vehicle-treated cells transfected with the GBS-flank library (Sgk flank library). (e) Same as (d) except for biological replicates of dexamethasone-treated cells (4h 1μM). (TIF) [file pgen.1007793.s001.tif]

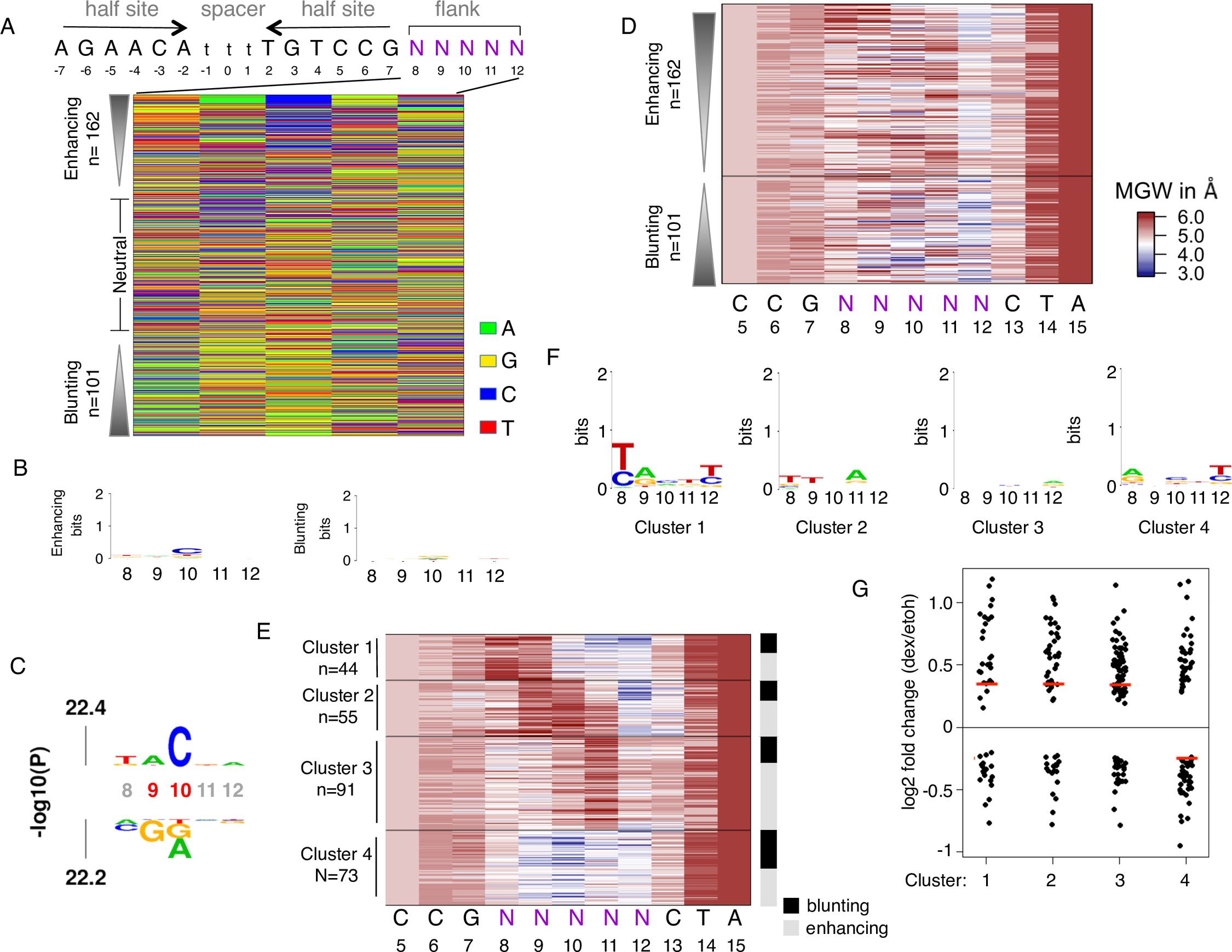

Supplement: S2 Fig — (a) Color chart summarizing the sequence at each variable position for flank variants ranked by their fold change in response to hormone treatment. (b) Consensus motif for (left) significantly enhancing and (right) blunting flank variants (c) kpLogo probability logo (activity logo) for flank variants depicting the p-values from Mann-Whitney U tests of whether GBS variants with a specific nucleotide at a given position are more (displayed above number indicating nucleotide position) or less (displayed below number indicating nucleotide position) active than other GBS variants. Positions with significant nucleotides (p < 0.001) are highlighted (red coordinates). (d) Predicted minor groove width (MGW) for significant enhancing and blunting flank variants of the Sgk GBS library ranked by their fold change in response to hormone treatment. (e) K-means clustering based on MGW for significantly enhancing and blunting flank variants. Right side: activating and blunting variants are highlighted in grey and black respectively. (f) Consensus sequence motif for clusters as indicated. (g) Log2 fold change upon dexamethasone treatment for each cluster as indicated. The synSTARR-seq activity for individual sequences is shown as black dots, the median for each group as a horizontal red line. (TIFF) [file pgen.1007793.s002.tiff]

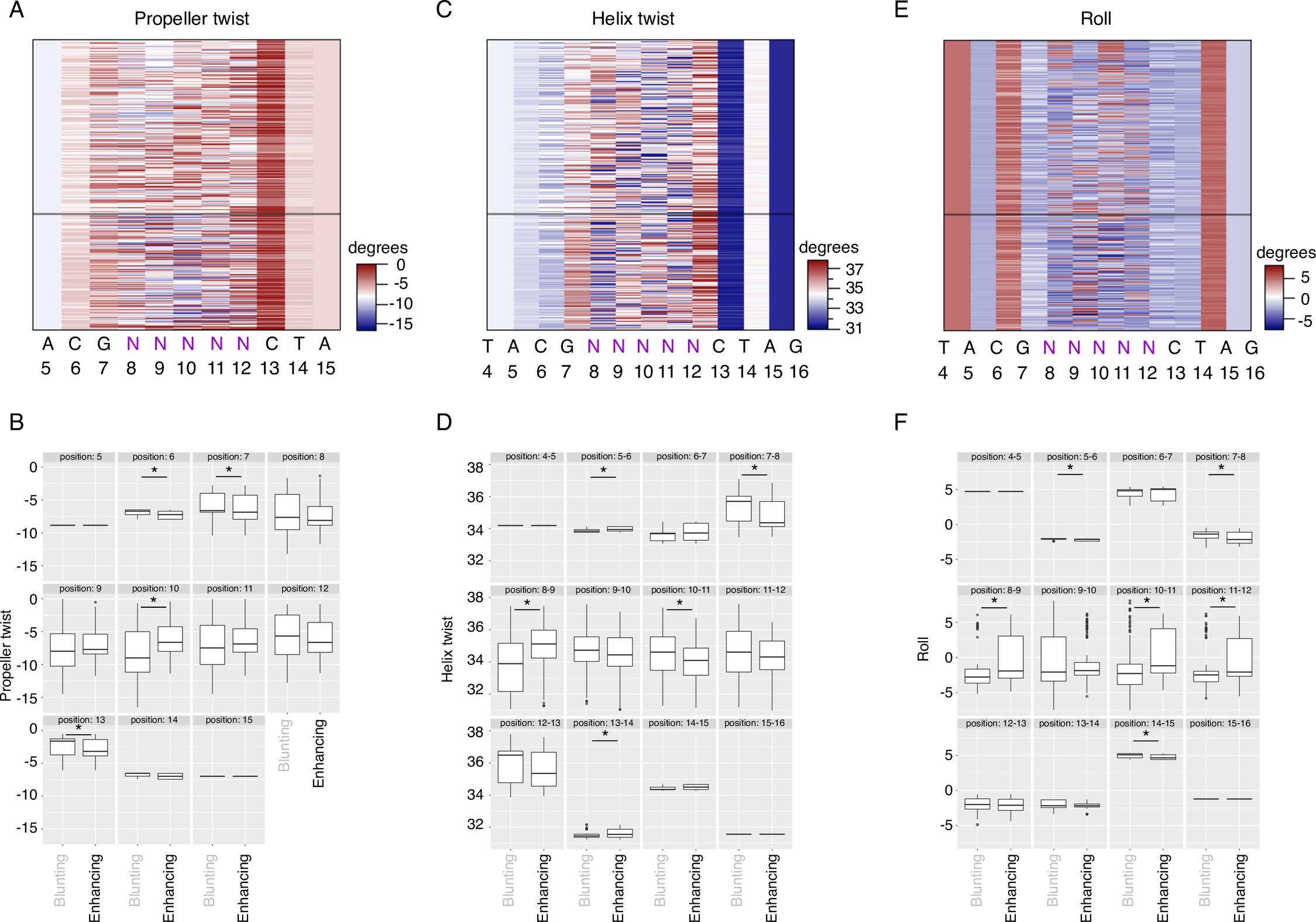

Supplement: S3 Fig — (a) Predicted Propeller twist for significant enhancing and blunting flank variants of the Cgt GBS library ranked by their fold change in response to hormone treatment. (b) Propeller twist for selected individual bases for significantly blunting (n = 189) and significantly enhancing (n = 125) flanks for the Cgt library. p-values were calculated using the Wilcoxon rank-sum test (*p < 0.01). (c) Same as (a) except that helix twist is shown. (d) same as (b) except that helix twist is shown. (e) Same as (a) except that roll is shown. (f) same as (b) except that roll is shown. (TIFF) [file pgen.1007793.s003.tiff]

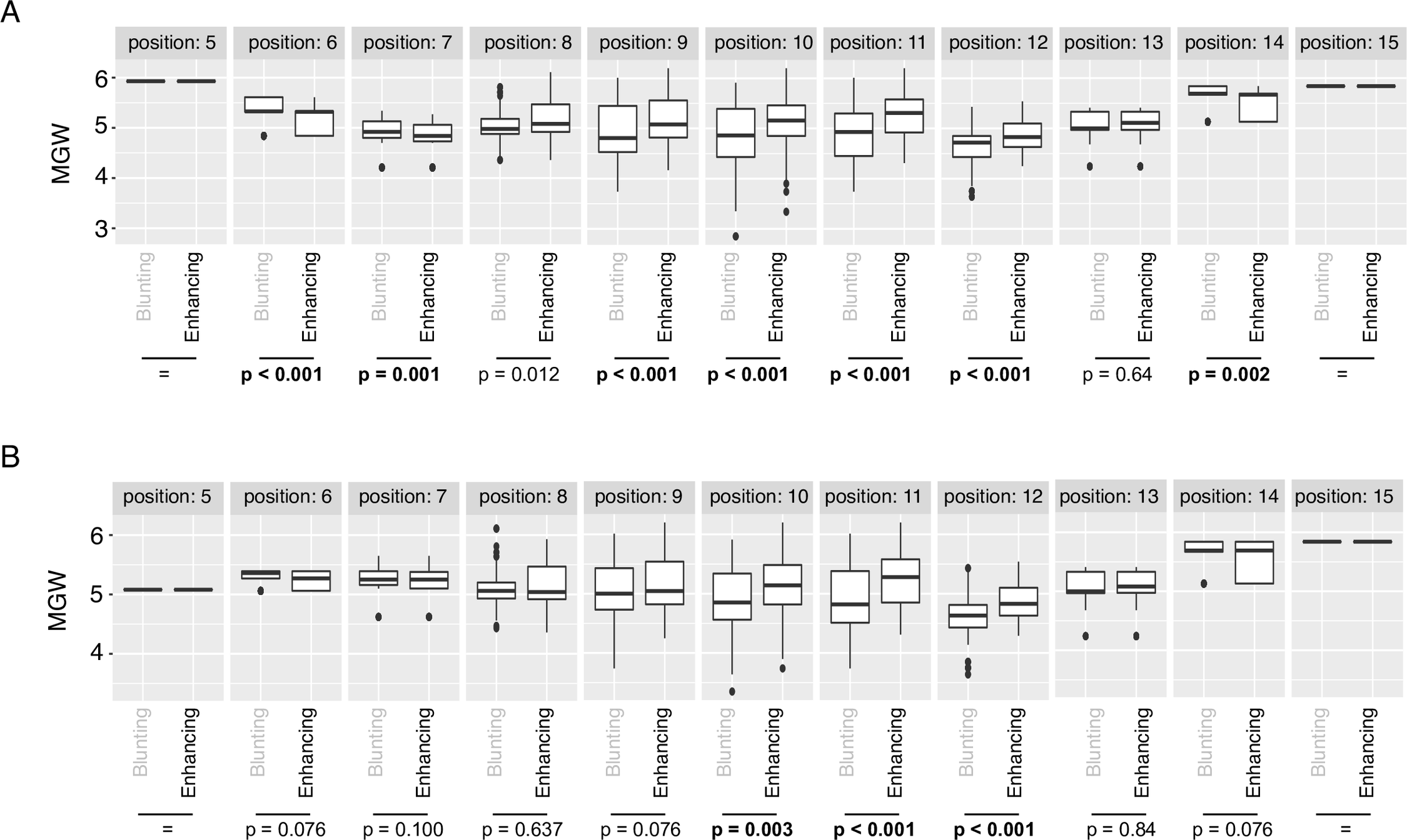

Supplement: S4 Fig — (a) Minor groove width (MGW) for selected individual bases for significantly blunting (n = 189) and significantly enhancing (n = 125) flanks for the Cgt library. p-values were calculated using the Wilcoxon rank-sum test. (b) Same as for (a) except for significantly blunting (n = 162) and significantly enhancing (n = 101) flanks of the Sgk flank library. (TIFF) [file pgen.1007793.s004.tiff]

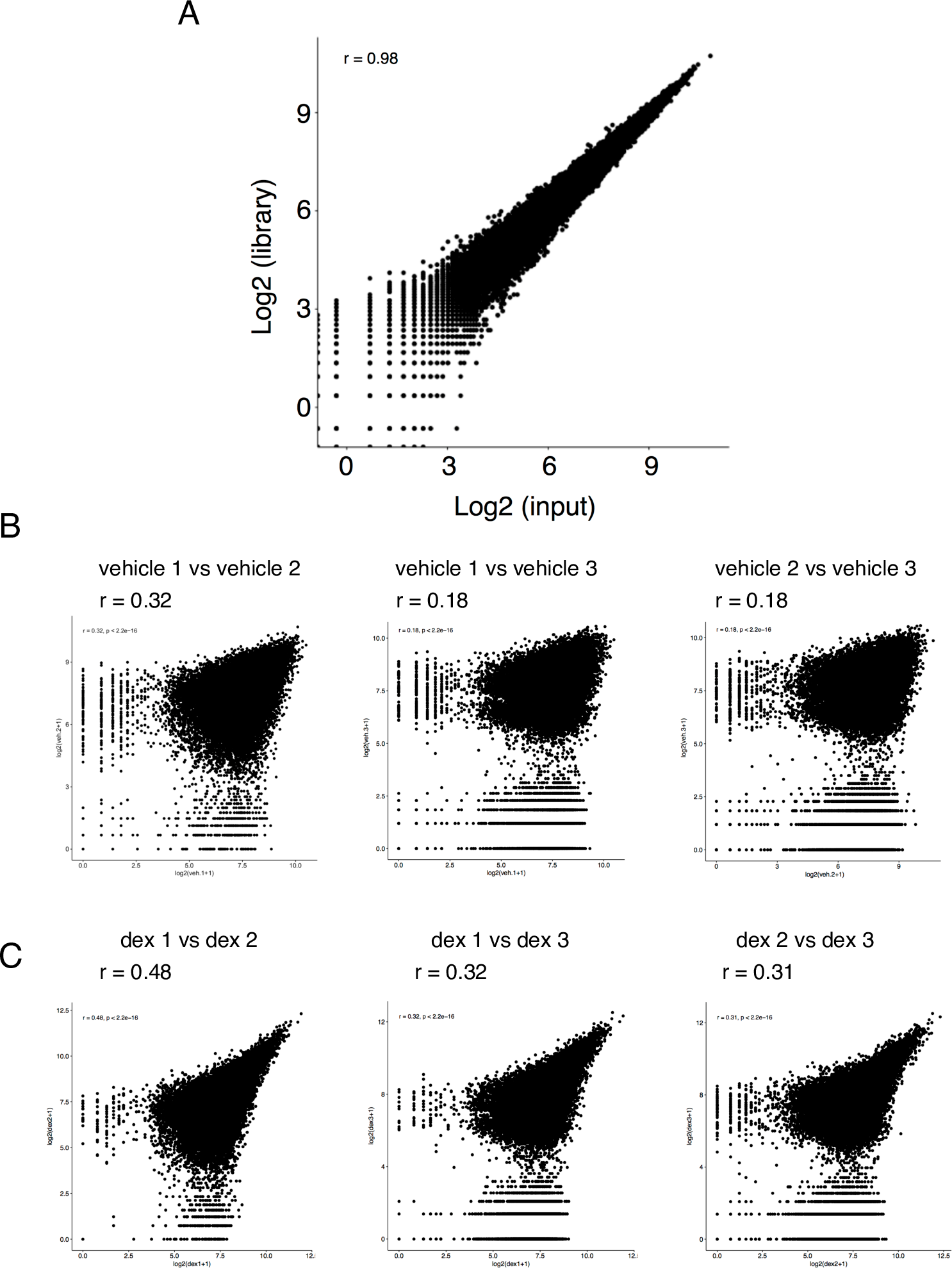

Supplement: S5 Fig — (a) Correlation plot between input library (library) and the plasmid library isolated from transfected U2OS-GR cells (input). (b) RNA-seq correlation plots for biological replicates of vehicle-treated cells. (c) Same as for (b) except for biological replicates of dexamethasone-treated cells (4h 1μM). (TIFF) [file pgen.1007793.s005.tiff]

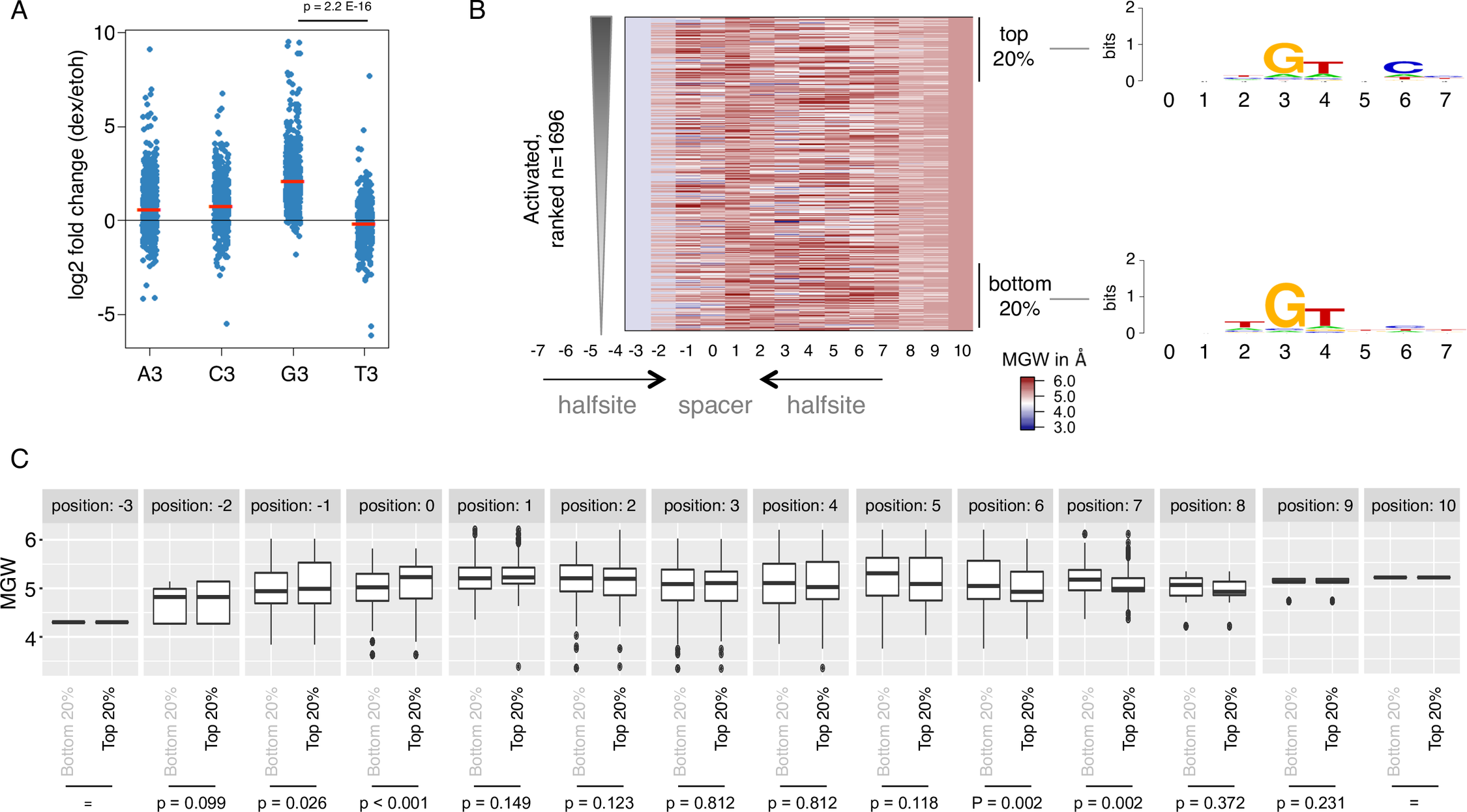

Supplement: S6 Fig — (a) Log2 fold change upon dexamethasone treatment for active GBS variants with either an A, C, G or T at position 3. Data for individual sequences that match consensus second half site at key positions 4 and 6 (exact match to AGAACATnnnXTnCn, with X either A,C,G or T) are shown as blue dots. Horizontal red lines show the average for each group. p-value was calculated using a Student’s t-test. (b) Left: Minor groove width (MGW) prediction for GBS variants ranked by activity. Right: Consensus motif for top 20% most active and bottom 20% least active GBS variants. (c) MGW for select individual bases comparing the top 20% most active and bottom 20% least active activated GBS variants. p-values were calculated using the Wilcoxon rank-sum test. (TIFF) [file pgen.1007793.s006.tiff]

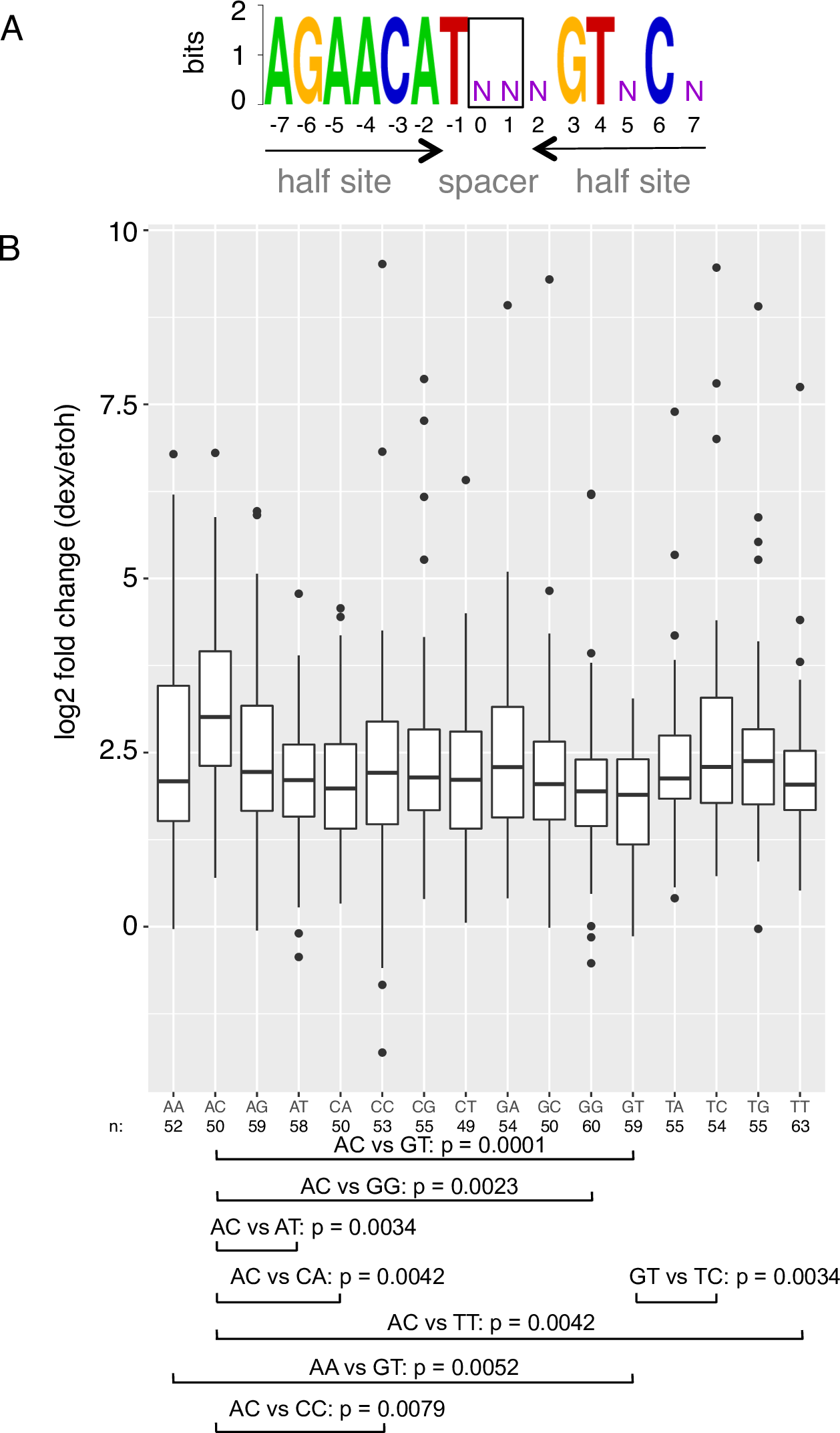

Supplement: S7 Fig — (a) Motif logo representing the sequence that was used to scan for GBS-matches in the half site library. Black box highlights the two positions in the spacer whose effects on GBS activity was assayed. (b) Boxplot of the log2 fold change upon treatment for 4 h with 1 μM dexamethasone for GBS matches with spacer variant as indicated. Center lines show the median. Spacer variants with significantly different activity levels (Benjamini-Hochberg corrected p-values calculated using a Student’s t-test < 0.01 are highlighted). (TIFF) [file pgen.1007793.s007.tiff]

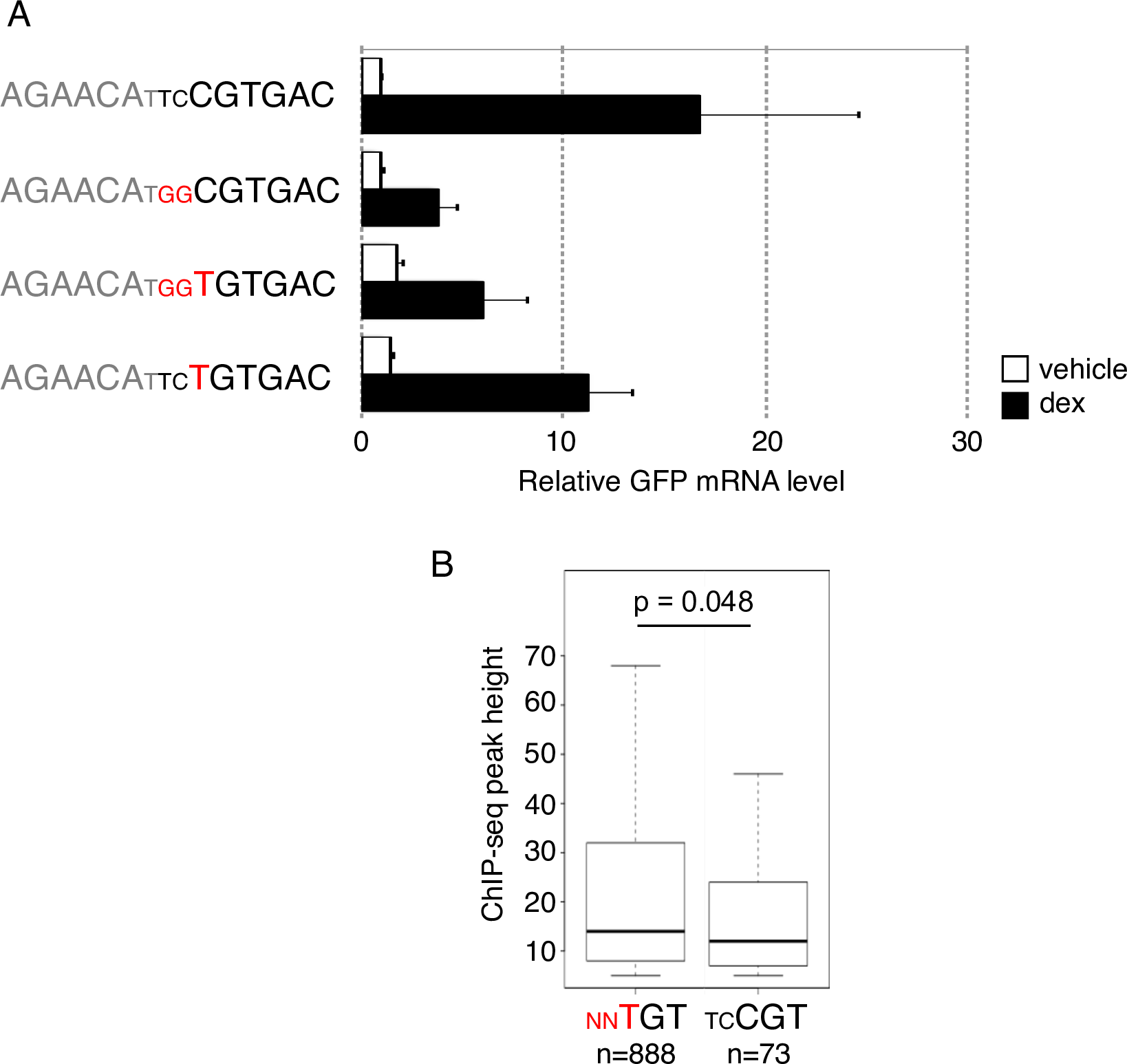

Supplement: S8 Fig — (a) Transcriptional activity of STARR-seq reporters containing candidate GBS variants as indicated. Relative RNA levels ± S.E.M. are shown for cells treated with ethanol vehicle and for cells treated overnight with 1 μM dexamethasone (n = 3). (b) Boxplot showing the peak-height for GR target genes with either a canonical GBS motif match (nnTGT) or a combi2 motif match (tcCGT). Center lines show the median, p value was calculated using a Wilcoxon rank-sum test. (TIFF) [file pgen.1007793.s008.tiff]

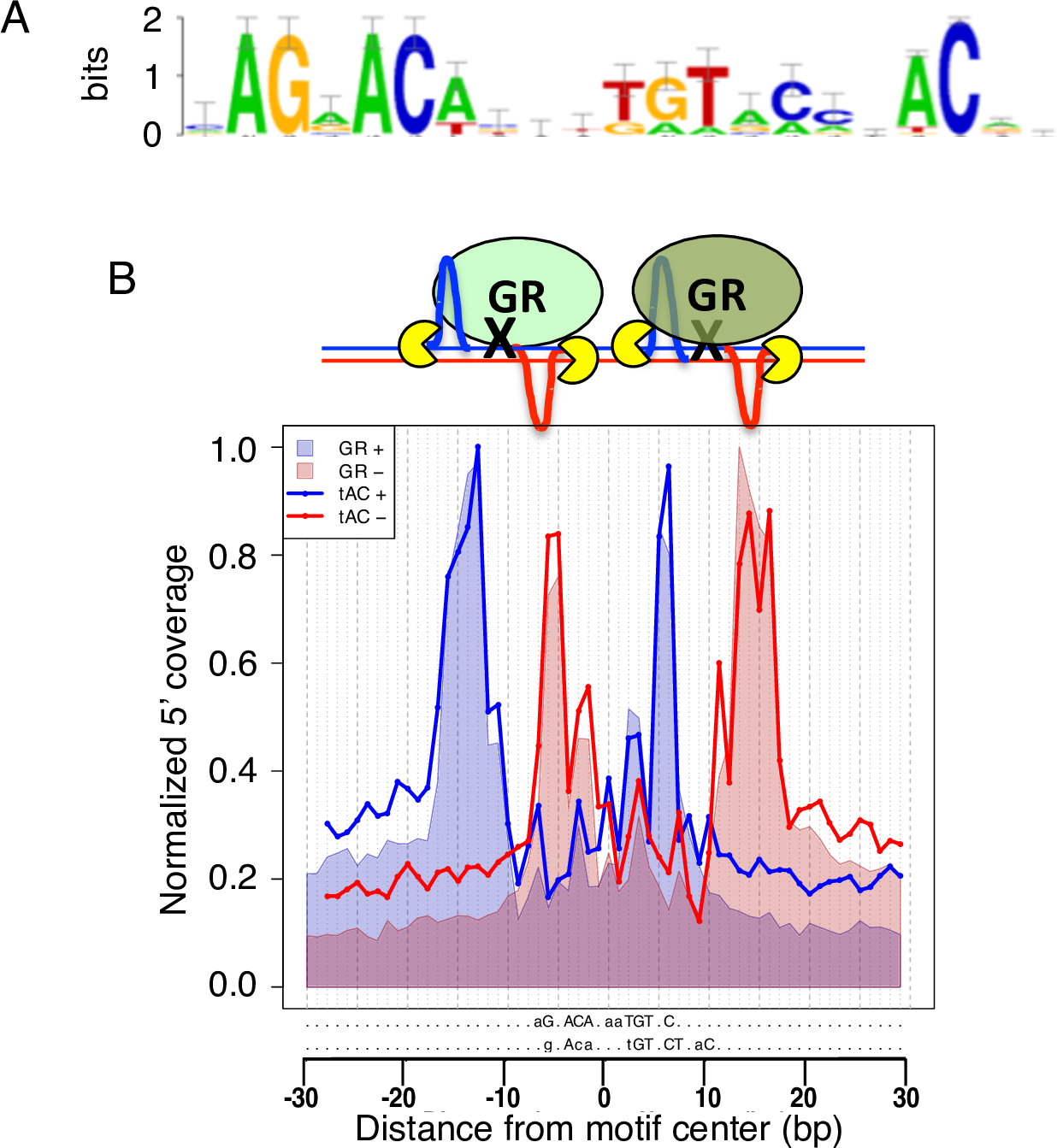

Supplement: S9 Fig — (a) Motif logo representing the positional weight matrix of highly active flank variants that was used to scan for motif-matches to generate the ChIP-exo footprint profile. (b) Alignment of the ChIP-exo footprint profiles for highly active flank variant matches (p value <0.0001; solid lines: blue: positive strand, red: negative strand) and for the conventional GBS motif (M00205; p value <0.0001; shaded areas; blue: positive strand, red: negative strand). (TIFF) [file pgen.1007793.s009.tiff]

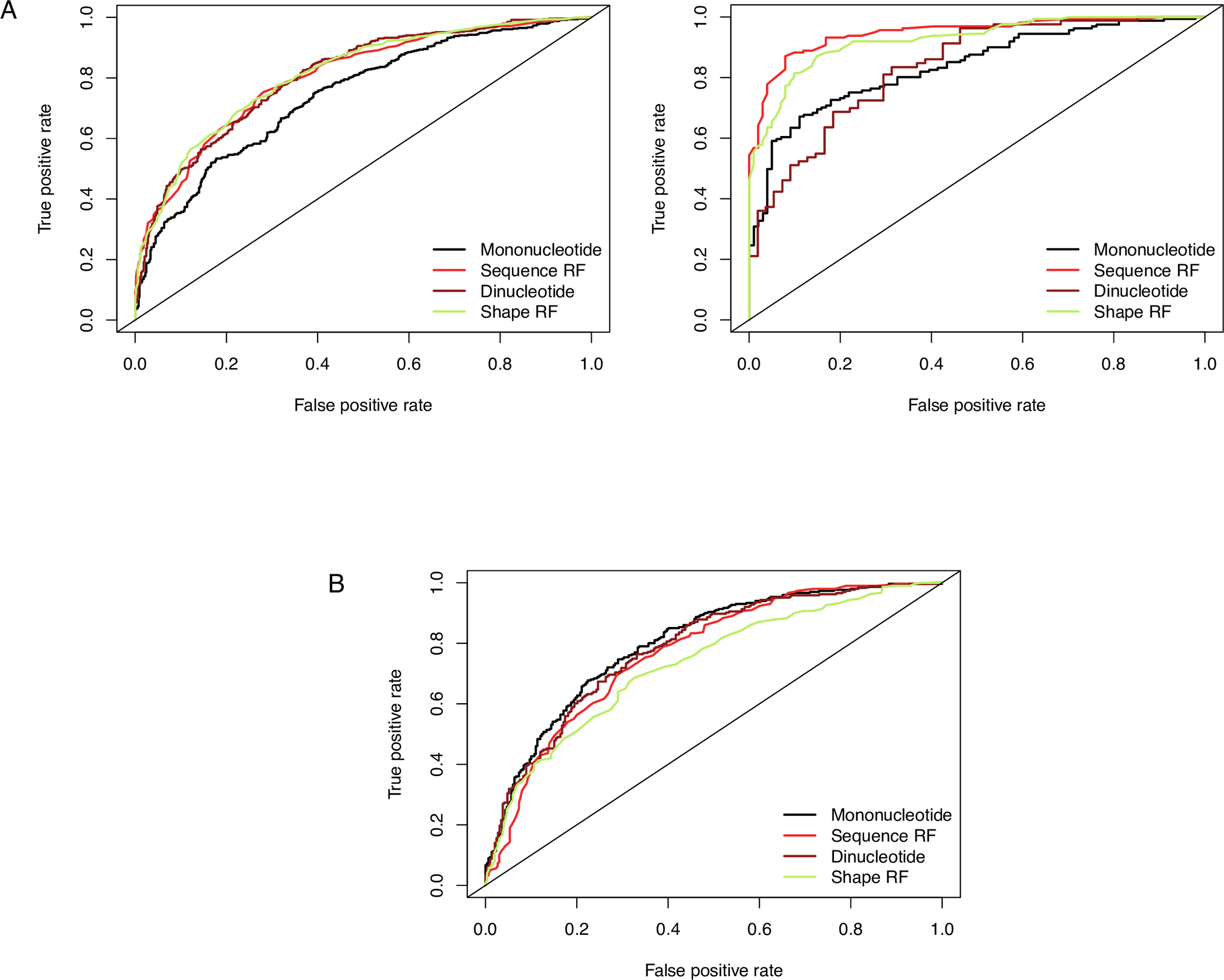

Supplement: S10 Fig — (a) ROC curves analyzing the ability of the models to distinguish between blunting and enhancing flank variants for (left) the Cgt flank library; (right) the Sgk flank library. Mononucleotide: Classifier based on mononucleotide frequencies within the two classes. Dinucleotide: Classifier constructed using dinucleotide frequencies. Sequence Random Forest (RF): Random Forest classifer trained and tested on coded nucleotide sequences. Shape Random Forest (RF): Random forest classifier based on predicted MGW. (b) Same as for (a) except that model and ROC curves where trained and assessed for their ability to discriminate between the top and bottom 20% significantly active GBS variants from the half site library. (TIFF) [file pgen.1007793.s010.tiff]
