# Supplementary material for: Synthetic STARR-seq reveals how DNA shape and sequence modulate transcriptional output and noise
Source: PLoS Genet. 2018 Nov 14;14(11):e1007793. doi: 10.1371/journal.pgen.1007793 (PMC6261644; doi:10.1371/journal.pgen.1007793)
Supplement: S1 Table — GBS (or scrambled GBS) underlined. (PDF) [file pgen.1007793.s011.pdf]

S1 Table: Inserts plasmids

GBS (or scrambled GBS) underlined

Fig. 1B:

No GBS (scramble)	CAGCGAAAGAAACTCCGTTGCCCGTCGCT
Genomic FKBP5-2	211bp region hg19Chr6: 35699789-35699999
FKBP5-2 GBS	CAGCGAAAGAACATCCTGTGCCCGTCGCT

Fig. 1D:

Blunting 1	CAGCGAAAGAACAtttTGTACGCATGTCT
Blunting 2	CAGCGAAAGAACAtttTGTACGATTTTCT
Blunting 3	CAGCGAAAGAACAtttTGTACGATTGGCT
Blunting 4	CAGCGAAAGAACAtttTGTACGCGACCCT
Blunting 5	CAGCGAAAGAACAtttTGTACGGGGGTCT
Neutral 1	CAGCGAAAGAACAtttTGTACGTCAGTCT
Neutral 2	CAGCGAAAGAACAtttTGTACGTCCATCT
Neutral 3	CAGCGAAAGAACAtttTGTACGGGCTACT
Neutral 4	CAGCGAAAGAACAtttTGTACGACCGTCT
Neutral 5	CAGCGAAAGAACAtttTGTACGCTTGTCT
Enhancing 1	CAGCGAAAGAACAtttTGTACGTACGTCT
Enhancing 2	CAGCGAAAGAACAtttTGTACGGACATCT
Enhancing 3	CAGCGAAAGAACAtttTGTACGATCGTCT
Enhancing 4	CAGCGAAAGAACAtttTGTACGTACGCCT
Enhancing 5	CAGCGAAAGAACAtttTGTACGTGTTCCT

Fig. 2D:

Enhancing	CAGCGAAAGAACAtttTGTACGTACGTCT
Neutral	CAGCGAAAGAACAtttTGTACGTCAGTCT
Blunting	CAGCGAAAGAACAtttTGTACGCATGTCT

Fig. 4C:

Negative1	CAGCGAAAGAACATCCTACAGCCGTCGCT
Negative2	CAGCGAAAGAACATGTCCGCATCGTCGCT
Negative3	CAGCGAAAGAACATGAGAAGGACGTCGCT
Negative4	CAGCGAAAGAACATTTCTCATTCGTCGCT
Weak1	CAGCGAAAGAACATTTTTTCCTCGTCGCT
Weak2	CAGCGAAAGAACATAGTGTCGTCGTCGCT
Weak3	CAGCGAAAGAACATTAACAAGGCGTCGCT
Weak4	CAGCGAAAGAACATTATGTTCTCGTCGCT
Strong1	CAGCGAAAGAACATGACGTACCCGTCGCT
Strong2	CAGCGAAAGAACATTGAGTCCCCGTCGCT
Strong3	CAGCGAAAGAACATTCCGTCTGCGTCGCT
Strong4	CAGCGAAAGAACATTCAGTACCCGTCGCT
Strong5	CAGCGAAAGAACATTGTGCACCCGTCGCT
Strong6	CAGCGAAAGAACATCGCGTCCTCGTCGCT
Strong7	CAGCGAAAGAACATTCCGTCCACGTCGCT
Strong8	CAGCGAAAGAACATTCCGTGACCGTCGCT

Fig. 5B:

123-TTCCGTCCA	CAGCGAAAGAACATTCCGTCCACGTCGCT
123-TGGCGTCCA	CAGCGAAAGAACATGGCGTCCACGTCGCT
123-TGGTGTCCA	CAGCGAAAGAACATGGTGTCCACGTCGCT
123-TTCTGTCCA	CAGCGAAAGAACATTCTGTCCACGTCGCT

Fig. S8A:

124-TTCCGTGAC	CAGCGAAAGAACATTCCGTGACCGTCGCT
124-TGGCGTGAC	CAGCGAAAGAACATGGCGTGACCGTCGCT
124-TGGTGTGAC	CAGCGAAAGAACATGGTGTGACCGTCGCT
124-TTCTGTGAC	CAGCGAAAGAACATTCTGTGACCGTCGCT

Fig. 6:

1 x FKBP5-2	CAGCGAAAGAACAgggTGTTCTCGTCGCT
3 x FKBP5-2	AGAACATCCTGTGCCgtacAGAACATCCTGTGCCTCGAAGAACATCCTGTGCCAGATCGGAA
**ETS1** + FKBP5-2	CAGCGAAAGAACATCCTGTGCCCGTCGCTAAG**ACAGGA**CCTAGTT
1xCGT	CAGCGAAAGAACAtttTGTACGCGTCGCT
1xFKBP5-2	CAGCGAAAGAACATCCTGTGCCCGTCGCT
1x Gilz	CAGCGAAAGAACAttgGGTTCCCGTCGCT
1xPal	CAGCGAAAGAACAaaaTGTTCTCGTCGCT
3xCgt	AGAACAtttTGTACGgaacAGAACAtttTGTACGTCGAAGAACAtttTGTACGAGATCGG
3xFKBP5-1	AGAACAgggTGTTCTgaacAGAACAgggTGTTCTTCGAAGAACAgggTGTTCTAGATCGG
3xGILZ	AGAACAttgGGTTCCgaacAGAACAttgGGTTCCTCGAAGAACAttgGGTTCCAGATCGG
3xPal	AGAACAaaaTGTTCTgaacAGAACAaaaTGTTCTTCGAAGAACAaaaTGTTCTAGATCGG
3xSgk	AGAACAtttTGTCCGgaacAGAACAtttTGTCCGTCGAAGAACAtttTGTCCGAGATCGG
**AP1** + FKBP5-2	CAGCGAAAGAACATCCTGTGCCCGTCGCTAAG**TGAGTCA**CCTAGTT
**SP1** + FKBP5-2	CAGCGAAAGAACATCCTGTGCCCGTCGCTAAG**CCCTCCCC**CCTAGTT
Combi2a	CAGCGAAAGAACATTCCGTCCACGTCGCT
Combi2b	CAGCGAAAGAACATTCCGTGACCGTCGCT
Cgt + tAC flank	CAGCGAAAGAACAtttTGTACGTACGTCT
