# Supplementary material for: Synthetic STARR-seq reveals how DNA shape and sequence modulate transcriptional output and noise
Source: PLoS Genet. 2018 Nov 14;14(11):e1007793. doi: 10.1371/journal.pgen.1007793 (PMC6261644; doi:10.1371/journal.pgen.1007793)
Supplement: S2 Table — (PDF) [file pgen.1007793.s012.pdf]

S2 Table: Oligos for EMSAs

Name:	fw:	rev:
TACGT flank	5'Cy5-CGCAAGAACATTTTGTACGTACGTCTAGA	TCTAGACGTACGTACAAAATGTTCTTGCG
CATGT flank	5'Cy5-CGCAAGAACATTTTGTACGCATGTCTAGA	TCTAGACATGCGTACAAAATGTTCTTGCG
TCAGT flank	5'Cy5-CGCAAGAACATTTTGTACGTCAGTCTAGA	TCTAGACTGACGTACAAAATGTTCTTGCG
ATTTTT flank	5'Cy5-CGCAAGAACATTTTGTACGATTTTCTAGA	TCTAGAAAATCGTACAAAATGTTCTTGCG
